# Optimal Cut-Off Values of the Positive Lymph Node Ratio and the Number of Removed Nodes for Patients Receiving Resection of Bronchopulmonary Carcinoids: A Propensity Score-Weighted Analysis of the SEER Database

**DOI:** 10.3389/fonc.2021.696732

**Published:** 2021-07-21

**Authors:** Qichen Chen, Mingxia Li, Pan Wang, Jinghua Chen, Hong Zhao, Jun Zhao

**Affiliations:** ^1^ Department of Hepatobiliary Surgery, National Cancer Center/National Clinical Research Center for Cancer/Cancer Hospital, Chinese Academy of Medical Sciences and Peking Union Medical College, Beijing, China; ^2^ Peking Union Medical College, Chinese Academy of Medical Sciences and Peking Union Medical College, Beijing, China; ^3^ Department of Thoracic Surgery, National Cancer Center/National Clinical Research Center for Cancer/Cancer Hospital, Chinese Academy of Medical Sciences and Peking Union Medical College, Beijing, China; ^4^ Key Laboratory of Gene Editing Screening and R & D of Digestive System Tumor Drugs, Chinese Academy of Medical Sciences and Peking Union Medical College, Beijing, China

**Keywords:** bronchopulmonary carcinoids, lymph node dissection, positive lymph node ratio, number of removed nodes, prognosis, inverse probability of treatment propensity-score weighting

## Abstract

**Background:**

Although lymph node dissection (LND) has been commonly used for patients with bronchopulmonary carcinoids (PCs), the prognostic values of the positive lymph node ratio (PLNR) and the number of removed nodes (NRN) remain unclear.

**Methods:**

Patients with resected PCs were identified in the Surveillance, Epidemiology, and End Results (SEER) database (2010–2015). The optimal cut-off values of the PLNR and NRN were determined by X-tile. The inverse probability of treatment weighting (IPTW) method was used to reduce the selection bias. IPTW-adjusted Kaplan-Meier curves and Cox proportional hazards models were used to compare the overall survival (OS) and cancer-specific survival (CSS) of patients in different PLNR and NRN groups.

**Results:**

The study included 1622 patients. The optimal cut-off values of the PLNR and NRN for survival were 13% and 13, respectively. In both Kaplan-Meier analysis and univariable Cox proportional hazards regression analysis before IPTW, a PLNR ≥13% was significantly associated with worse OS (HR = 3.364, P<0.001) and worse CSS (HR = 7.874, P<0.001). These findings were corroborated by the IPTW-adjusted Cox analysis OS (HR = 2.358, P = 0.0275) and CSS (HR = 8.190, P<0.001) results. An NRN ≥13 was not significantly associated with worse OS in either the Kaplan-Meier or Cox analysis before or after IPTW adjustment. In the Cox proportional hazards analysis before and after IPTW adjustment, an NRN ≥13 was significantly associated with worse CSS (non-IPTW: HR = 2.216, P=0.013; IPTW-adjusted: HR = 2.162, P=0.024).

**Conclusion:**

A PLNR ≥13% could predict worse OS and CSS in patients with PCs and might be an important complement to the present PC staging system. Extensive LND with an NRN ≥13 might have no therapeutic value for OS and may even have an adverse influence on CSS. Its application should be considered on an individual basis.

## Introduction

Bronchopulmonary carcinoids (PCs) are rare neuroendocrine tumors comprising 20% to 30% of all neuroendocrine tumors (NETs) and accounting for approximately 1% to 2% of lung malignancies, with an estimated annual incidence of 0.2-2/100 000 persons/year (1). PCs are subdivided into 2 main groups, well-differentiated typical carcinoid (TC) and the less common moderate-differentiated atypical carcinoid (AC). The incidence of PCs has been increasing over the past 30 years, which might represent a genuine overall increase or result from increased awareness and improved diagnostic techniques (2, 3).

Surgery is an option for the treatment of PCs and aims to remove the tumor while preserving as much lung tissue as possible (4, 5). Lymph node dissection (LND) or sampling has been commonly applied during PC surgery. The National Comprehensive Cancer Network (NCCN), European Neuroendocrine Tumor Society (ENETS), and Commonwealth Neuroendocrine Tumor Research Collaboration and the North American Neuroendocrine Tumor Society (NANETS/CommNETs) guidelines recommend mediastinal LND or sampling accompanied by lobectomy for localized/locoregionally resectable PCs (4). Additionally, systematic LND is recommended by the European Society for Medical Oncology (ESMO), as lymph node involvement may be observed in up to 27% of TCs and in up to 47% of ACs (6). LND can help detect occult lymph node metastasis and thus evaluate prognosis, as lymph node involvement was suggested to be an independent predictor of worse survival (7). Although LND is currently accepted as the most accurate and reliable staging procedure for the detection of lymph node involvement, the optimal LND extent, which could be assessed by the number of removed nodes (NRN), remains unclear (8). Extensive LND might provide more information and potential therapeutic benefits, but the advantages might be offset by an increased risk for postoperative or other complications. Additionally, recent evidence indicated that the positive lymph node ratio (PLNR), which was defined as the ratio of positive to examined lymph nodes, is a significant prognostic determinant in other NETs, including gastric, small intestinal and pancreatic NETs (9–11).

Therefore, to further evaluate the potential prognostic value of PLNR and determine the optimal LND extent, we performed a retrospective study using the Surveillance, Epidemiology, and End Results (SEER) database. Inverse probability of treatment propensity-score weighting (IPTW) was employed to reduce the selection bias.

## Method

### Database

The Surveillance, Epidemiology, and End Results (SEER) database is a population-based database covering approximately 34.6% of the US population. The SEER registries collect data from 18 geographically diverse populations that represent rural, urban, and regional populations and include patient demographics, primary tumor site, tumor morphology, stage at diagnosis, and first course of treatment. These variables were included according to their clinical significance and previous research evidences that they might be confounders potentially influencing the prognosis (12, 13). The SEER registries follow up with patients to obtain vital status information. We identified patients with typical and atypical pulmonary carcinoid tumors diagnosed between 2010 and 2016 using SEER*Stat software (version 8.3.8). According to the International Classification of Disease for Oncology (3rd ed.) (ICD-O-3), histology codes 8240 (typical carcinoid) and 8249 (atypical carcinoid) were selected. The following primary site records were involved: C34.0-C34.3, C34.8, and C34.9.

### Patients

**Figure 1:**
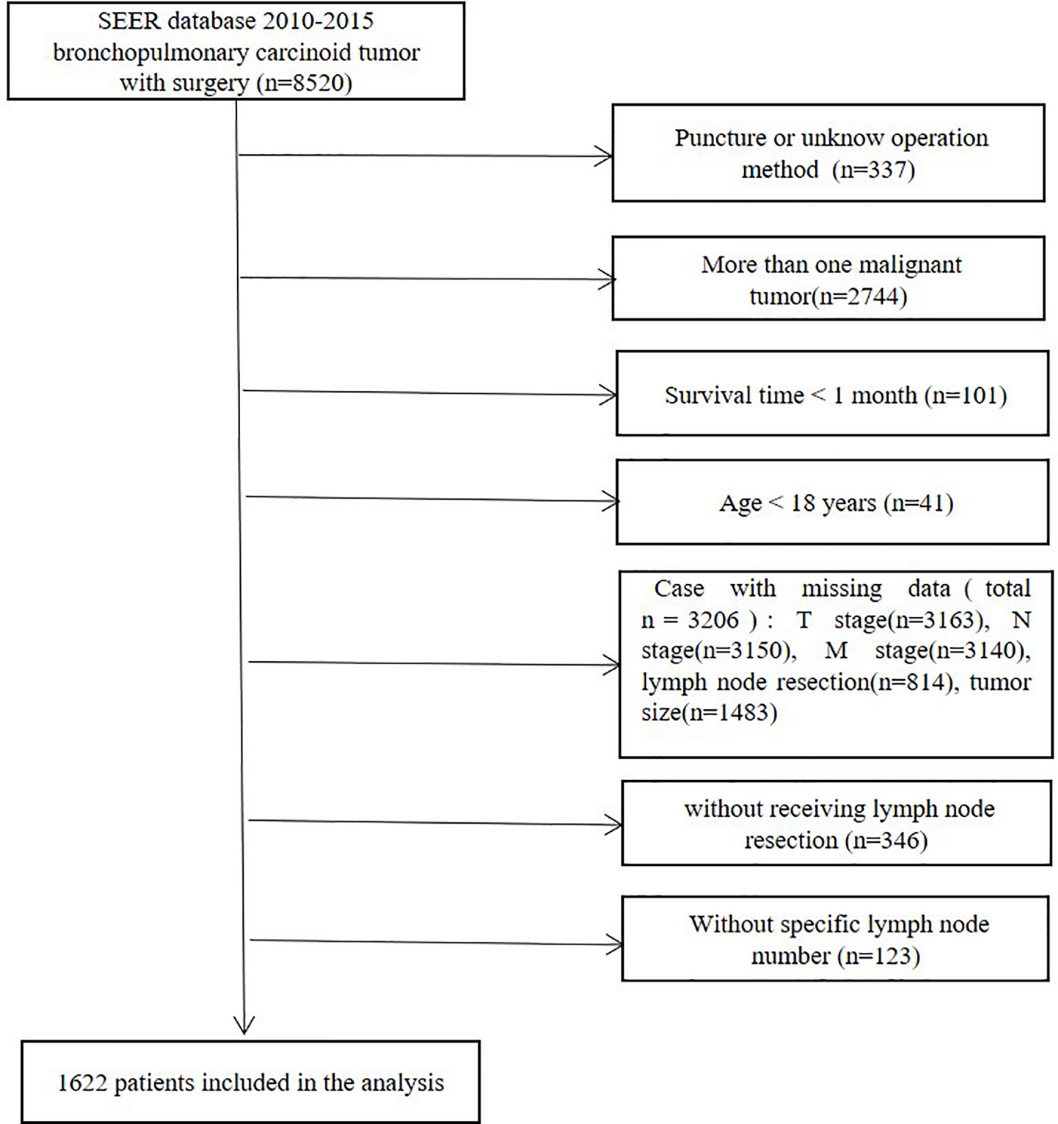
Flow diagram for the selection of bronchopulmonary carcinoid tumors included in the final analyses of this study.

The patient-level details were retrieved by SEER*Stat version 8.3.8 software. Patients were uniformly reviewed and staged according to the 7th edition of the TNM classification system (14). The inclusion criteria were as follows (**Figure 1**): (1) pathological bronchopulmonary carcinoid tumor and (2) surgery. The exclusion criteria were as follows: (1) more than one malignant tumor; (2) pathology of lung puncture or unknown operation method; (3) survival time < 1 month; (4) age < 18 years; (5) no lymph node resection; (6) no specific lymph node number; and (7) missing or incomplete patient information. The end points of our study were overall survival (OS) and cancer-specific survival (CSS). The following patient-related factors were included: age at diagnosis, year of diagnosis, sex, marital status and death status. Tumor-related factors included primary site, tumor size, histology, laterality, SEER stage, T stage, N stage and M stage. Treatment-related factors include LND, number of lymph nodes examined, positive lymph node number, surgical approach, chemotherapy and radiation treatment.

All patients included were diagnosed between 2010 and 2015. Initially, a total of 8520 patients with bronchopulmonary carcinoid tumors who received surgery were identified from the SEER 18 database. Patients who had lung puncture or an unknown operation method (n=337) were omitted. Patients were also excluded if they had more than one malignant tumor (n=2744), a survival time of <1 month (n=101) or if they were aged <18 years (n=41). Patients with missing data (total n=3206), including T stage (n=3163), N stage (n=3150), M stage (n=3140), lymph node resection (n=814) and tumor size (n=1483) data, were disqualified. Patients without lymph node resection or without available specific lymph node numbers were excluded. The final study group consisted of 1622 patients (**Figure 1**).

### Statistical Analysis

The PLNR was defined as the ratio of positive to examined lymph nodes. The extent of LND was assessed by the NRN. The optimal cut-off values of PLNR (13%) and NRN (13) were determined by X-tile software version 3.6.1 (Yale University) according to survival. The baseline characteristics between patients with PLNR ≥13% and patients with PLNR <13% were compared. The NRN ≥13 group was also compared with NRN <13 group. The balance in covariates was assessed by using the standardized difference (SD) approach. An SD of 0.1 denotes a meaningful imbalance in the factors between the two groups. The observed differences in the baseline covariates between the two intervention groups were adjusted by the IPTW method to eliminate selection bias (15). Multivariable logistic regression models were established to predict PLNR and NRN level. The adjusted Kaplan-Meier curves and log-rank test based on the non-IPTW and IPTW populations were used to compare OS and CSS between the PLNR ≥13% group and the PLNR <13% group. Cox proportional hazards model analyses the hazard, which is an instantaneous failure rate as a function of time. Coefficients are estimated for each variable and converted to hazard ratios (HR) for time to event outcomes (16). Univariable Cox proportional hazards models were used to estimate the non-IPTW and IPTW-adjusted HR of the PLNR ≥13% group versus the PLNR <13% group. The same analyses were applied to compare the NRN ≥13 group with the NRN <13 group. Data were analyzed using SPSS (version 25.0; IBM, Armonk, NY) and R software (version 3.6.0). A p value of <0.05 was considered statistically significant.

## Results

### Patient Characteristics

**Figure 2:**
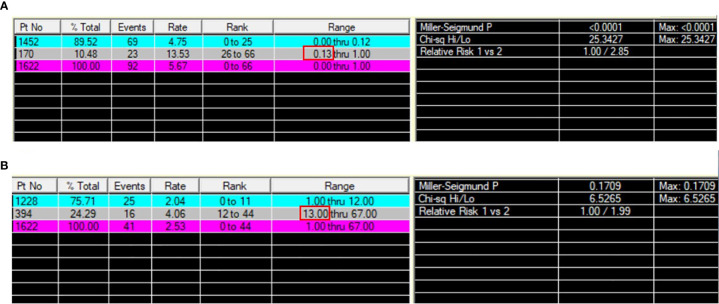
Optimal cut-off value analyses of PLNR **(A)** and NRN **(B)** by X-tile.

In total, 1622 patients met the study inclusion criteria. 32.6% of patients were male. 53.3% of patients were under the age of 60 (including 60) and 18.1% were older than 70. 60.1% of patients were married. 41.7%, 19.5%, 31.3%, and 7.5% of the primary sites were distributed in lower, middle, upper lobes and other locations, and 40.9% of them were at left. The ACs accounts for 12.5% of patients. 70.3%, 25.4%, and 4.3% of patients were classified into localized, reginal, and distant SEER Stage. 62.6%, 25.6%, 8.6% and 3.1% of patients were at T1, T2, T3, and T4 stage, respectively. N0, N1, and N2+N3 stage were observed in 85.0%, 10.0%, and 4.9% of patients, respectively. 2.0% of patients were at M1 stage. Most patients received lobectomy (81.9%), 8.9%, 4.9%, and 4.3% of patients received wedge resection, pneumonectomy and segmental resection, respectively. The tumor size was smaller than 2.5 cm in 60.4% of patients. 3.1% and 2.1% of patients received chemotherapy and radiation. The PLNR ranged from 0 to 100%. The optimal cut-off value of the PLNR for survival was 13% as determined by X-tile (**Figure 2**). A total of 161 patients presented with PLNR ≥13%, and 1461 patients presented with PLNR <13%. The median NRN was 7 (95% CI 4-12). The optimal cut-off value of the NRN for survival was 13 by as determined by X-tile (**Figure 2**). A total of 394 patients presented with NRN ≥13, and 1228 patients presented with NRN <13. The baseline characteristics of eligible patients before and after propensity score matching, stratified by the PLNR and NRN, are listed in **Tables 1**, **2**.

**Table 1:** Selected baseline characteristics between PLNR<0.13 and PLNR ≥0.13 before and after weighting.

Factor	Unweighted Study Population, No. (%)			Weighted Study Population, %		
	PLNR < 0.13 n=1461	PLNR ≥ 0.13 n = 161	Standardized difference	PLNR < 0.13	PLNR ≥ 0.13	Standardized difference
**Sex**						
Male	468 (32.0)	60 (37.3)	0.110	40.1 (38.3)	42.8 (38.8)	0.011
**Age**			0.115			0.018
≤60	770 (52.7)	94 (58.4)		65.6 (62.5)	69.5 (63.0)	
61-70	424 (29.0)	41 (25.5)		23.4 (22.4)	23.9 (21.6)	
>70	267 (18.3)	26 (16.1)		15.9 (15.1)	17.0 (15.4)	
**Marital Status**						0.005
Married	874 (59.8)	101 (62.7)	0.060	63.1 (60.1)	66.6 (60.4)	
**Primary Site**			0.089			0.012
Lower lobe	615 (42.1)	62 (38.5)		41.7 (39.8)	43.5 (39.4)	
Middle lobe	283 (19.4)	33 (20.5)		20.3 (19.4)	21.2 (19.2)	
Upper lobe	452 (30.9)	55 (34.2)		35.1 (33.4)	37.5 (34.0)	
Other	111 (7.6)	11 (6.8)		7.8 (7.4)	8.2 (7.5)	
**Histology**			0.591			0.018
Atypical carcinomas	148 (10.1)	54 (33.5)		27.4 (26.1)	28.0 (25.3)	
**Laterality**			0.031			0.039
Right+other	866 (59.3)	93 (57.8)		62.1 (59.2)	63.2 (57.2)	
**SEER Stage**			2.691			0.002
Localized	1140 (78.0)	0 (0.0)		0.0 (0.0)	0.0 (0.0)	
Regional	265 (18.1)	147 (91.3)		97.3 (92.7)	102.3 (92.7)	
Distant	56 (3.8)	14 (8.7)		7.6 (7.3)	8.1 (7.3)	
**7th T Stage**			0.458			0.036
T1	947 (64.8)	69 (42.9)		33.6 (32.1)	37.0 (33.5)	
T2	355 (24.3)	60 (37.3)		48.3 (46.0)	49.1 (44.4)	
T3	115 (7.9)	25 (15.5)		19.8 (18.9)	20.8 (18.8)	
T4	44 (3.0)	7 (4.3)		3.2 (3.0)	3.5 (3.2)	
**7th M Stage**			0.241			0.001
M1	23 (1.6)	10 (6.2)		6.0 (5.7)	6.3 (5.7)	
**Surgery**			0.319			0.033
Wedge Resection	140 (9.6)	5 (3.1)		3.2 (3.1)	3.1 (2.8)	
Segmental Resection	64 (4.4)	5 (3.1)		3.0 (2.8)	3.4 (3.0)	
Lobectomy	1192 (81.6)	137 (85.1)		90.9 (86.6)	94.9 (86.0)	
Pneumonectomy	65 (4.4)	14 (8.7)		7.9 (7.5)	9.1 (8.2)	
**Tumor Size**≥2.5 cm	542 (37.1)	100 (62.1)	0.517	65.4 (62.4)	65.7 (59.5)	0.058
**Chemotherapy**	17 (1.2)	34 (21.1)	0.669	7.8 (7.4)	8.6 (7.8)	0.015
**Radiation**	13 (0.9)	21 (13.0)	0.492	4.5 (4.3)	5.6 (5.1)	0.038

PLNR, positive lymph node ratio; NRN, number of removed nodes.

**Table 2:** Selected baseline characteristics between NRN<13 and NRN ≥ 13 before and after weighting.

Factor	Unweighted Study Population, No. (%)			Weighted Study Population, %		
	NRN < 13 n = 1228	NRN ≥ 13 n = 394	Standardized difference	NRN < 13	NRN ≥ 13	Standardized difference
**Sex**			0.112			0.012
Male	384 (31.3)	144 (36.5)		142.9 (37.1)	140.2 (36.6)	
**Age**			0.057			0.017
≤60	648 (52.8)	216 (54.8)		207.8 (54.0)	208.4 (54.4)	
61-70	352 (28.7)	113 (28.7)		109.7 (28.5)	110.5 (28.8)	
>70	228 (18.6)	65 (16.5)		67.2 (17.5)	64.5 (16.8)	
**Marital Status**						
Married	732 (59.6)	243 (61.7)	0.042	235.3 (61.2)	235.9 (61.5)	0.008
**Primary Site**			0.296			0.007
Lower lobe	508 (41.4)	169 (42.9)		170.0 (44.2)	168.5 (43.9)	
Middle lobe	265 (21.6)	51 (12.9)		50.6 (13.1)	51.0 (13.3)	
Upper lobe	381 (31.0)	126 (32.0)		123.0 (32.0)	123.2 (32.1)	
Other	74 (6.0)	48 (12.2)		41.1 (10.7)	40.8 (10.6)	
**Histology**			0.098			0.008
Atypical carcinomas	143 (11.6)	59 (15.0)		55.1 (14.3)	56.0 (14.6)	
**Laterality**						
Right+other	736 (59.9)	223 (56.6)	0.068	217.6 (56.6)	217.8 (56.8)	0.005
**SEER Stage**			0.209			0.021
Localized	892 (72.6)	248 (62.9)		250.9 (65.2)	246.7 (64.3)	
Regional	288 (23.5)	124 (31.5)		116.1 (30.2)	117.8 (30.7)	
Distant	48 (3.9)	22 (5.6)		17.7 (4.6)	18.9 (4.9)	
**7th T Stage**			0.170			0.018
T1	792 (64.5)	224 (56.9)		225.5 (58.6)	222.5 (58.0)	
T2	297 (24.2)	118 (29.9)		114.1 (29.7)	114.0 (29.7)	
T3	105 (8.6)	35 (8.9)		31.0 (8.1)	32.6 (8.5)	
T4	34 (2.8)	17 (4.3)		14.1 (3.7)	14.3 (3.7)	
**7th N Stage**			0.214			0.010
N0	1066 (86.8)	313 (79.4)		310.1 (80.6)	307.8 (80.3)	
N1	114 (9.3)	49 (12.4)		46.8 (12.2)	47.0 (12.2)	
N2+N3	48 (3.9)	32 (8.1)		27.8 (7.2)	28.6 (7.5)	
**7th M Stage**			0.067			0.019
M1	22 (1.8)	11 (2.8)		7.7 (2.0)	8.7 (2.3)	
**Surgery**			0.471			0.009
Wedge Resection	136 (11.1)	9 (2.3)		9.2 (2.4)	9.0 (2.3)	
Segmental Resection	59 (4.8)	10 (2.5)		10.2 (2.6)	10.0 (2.6)	
Lobectomy	995 (81.0)	334 (84.8)		332.9 (86.5)	332.9 (86.8)	
Pneumonectomy	38 (3.1)	41 (10.4)		32.5 (8.4)	31.5 (8.2)	
**Tumor Size**≥2.5 cm	454 (37.0)	188 (47.7)	0.219	178.1 (46.3)	180.2 (47.0)	0.014
**Chemotherapy**	37 (3.0)	14 (3.6)	0.030	13.0 (3.4)	12.9 (3.4)	<0.001
**Radiation**	28 (2.3)	6 (1.5)	0.055	6.3 (1.6)	6.0 (1.6)	0.006

PLNR, positive lymph node ratio; NRN, number of removed nodes.

**Figure 3:**
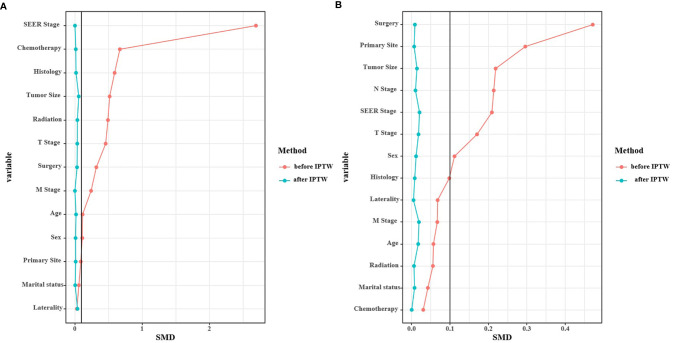
Standardized mean difference (SMD) of PLNR ≥ 13% *vs*. PLNR < 13% **(A)** and NRN ≥ 13 *vs*. NRN < 13 **(B)** before and after IPTW.

In the multivariable logistic regression analysis, atypical carcinomas (OR=3.869, 95% CI: 2.645-5.660, *P*<0.001), M1 stage (OR=4.315, 95% CI: 1.899-9.804, *P*<0.001) and tumor size ≥ 2.5 cm (OR=2.393, 95% CI: 1.693-3.382, *P*<0.001) were significantly associated with the likelihood of a PLNR ≥ 13% (**Table 3**). The results of the multivariable logistic regression analysis before IPTW adjustment demonstrated that N2+N3 stage (OR=1.983, 95% CI: 1.225-3.211, *P=*0.005), lobectomy (OR=4.886, 95% CI: 2.453-9.730, *P*<0.001) and pneumonectomy (OR=12.447, 95% CI: 5.479-28.276, *P*<0.001) were all significantly associated with NRN ≥ 13, and a middle-lobe primary site was significantly associated with NRN < 13 (OR=0.573, 95% CI: 0.403-0.815, *P=*0.002) (**Table 3**). After IPTW adjustment, the SD for all characteristics was less than 0.1, indicating that the weighted population in the PLNR ≥13% *vs.* PLNR <13% and NRN ≥ 13 *vs.* NRN < 13 groups were subsequently comparable (**Table 1** and **Figure 3**).

**Table 3:** Multivariable logistic regression model predicting PLNR level and NRN level in unweighted study population.

	PLNR < 0.13 *vs*. PLNR ≥ 0.13				NRN < 13 *vs*. NRN ≥ 13			
Factor	Univariate analysis		Multivariate analysis		Univariate analysis		Multivariate analysis	
	*P*	OR (95%CI)	*P*	OR (95%CI)	*P*	OR (95%CI)	*P*	OR (95%CI)
**Sex**								
Male	0.179	1.260 (0.899-1.767)			0.052	1.266 (0.998-1.606)		
**Age**								
≤ 60		Reference				Reference		
61-70	0.236	0.792 (0.539-1.165)			0.778	0.963 (0.741-1.251)		
>70	0.331	0.798 (0.506-1.259)			0.332	0.855 (0.624-1.173)		
**Marital Status**								
Married	0.474	1.131 (0.808-1.582)			0.466	1.090 (0.864-1.376)		
**Primary Site**								
Lower lobe		Reference				Reference		
Middle lobe	0.522	1.157 (0.741-1.805)			0.002	0.578 (0.409-0.818)	0.002	0.573 (0.403-0.815)
Upper lobe	0.335	1.207 (0.823-1.770)			0.965	0.994 (0.762-1.297)	0.917	0.986 (0.750-1.296)
Other	0.960	0.983 (0.502-1.926)			0.001	1.950 (1.303-2.917)	0.063	1.497 (0.978-2.290)
**Histology**								
Atypical carcinomas	0.000	4.477 (3.097-6.473)	0.000	3.869 (2.645-5.660)	0.082	1.336 (0.964-1.853)		
**Laterality**								
Right+other	0.711	0.940 (0.676-1.307)			0.241	0.872 (0.693-1.097)		
**SEER Stage**								
Localized		Reference				Reference		
Regional	0.986	>99 (0->99)			0.001	1.549 (1.202-1.995)		
Distant	0.987	>99 (0->99)			0.061	1.649 (0.976-2.784)		
**7th T Stage**								
T1		Reference				Reference		
T2	0.00	2.320 (1.607-3.348)			0.010	1.405 (1.083-1.821)		
T3	0.000	2.984 (1.815-4.903)			0.433	1.179 (0.782-1.776)		
T4	0.067	2.183 (0.948-5.028)			0.063	1.768 (0.969-3.224)		
**7th N Stage**								
N0		Reference				Reference		
N1	0.984	>99 (0->99)			0.037	1.464 (1.024-2.093)	0.180	1.283 (0.891-1.848)
N2+N3	0.983	>99 (0->99)			0.001	2.271 (1.427-3.614)	0.005	1.983 (1.225-3.211)
**7th M Stage**								
M1	0.000	4.141 (1.934-8.863)	0.000	4.315 (1.899-9.804)	0.225	1.574 (0.757-3.276)		
**Surgery**								
Wedge Resection		Reference				Reference		
Segmental Resection	0.229	2.187 (0.612-7.824)			0.053	2.561 (0.990-6.629)	0.079	2.356 (0.906-6.127)
Lobectomy	0.012	3.218 (1.296-7.990)			0.000	5.072 (2.555-10.072)	0.000	4.886 (2.453-9.730)
Pneumonectomy	0.001	6.031 (2.084-17.453)			0.000	16.304 (7.281-36.509)	0.000	12.447 (5.479-28.276)
**Tumor Size**≥2.5 cm	0.000	2.780 (1.987-3.888)	0.000	2.393 (1.693-3.382)	0.000	1.556 (1.237-1.957)		

PLNR, positive lymph node ratio; NRN, number of removed nodes.

### Survival Analyses Before and After IPTW Adjustment: PLNR ≥ 13% *vs*. PLNR < 13%

**Figure 4:**
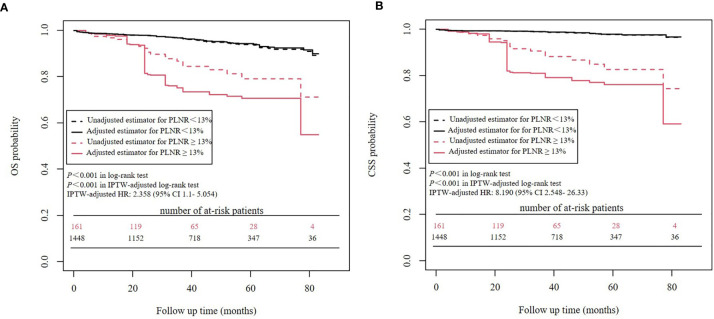
Survival analysis of PLNR before and after IPTW. **(A)** OS analysis **(B)** CSS analysis.

Kaplan-Meier analysis showed that a PLNR ≥ 13% was significantly associated with worse OS (*P*<0.001) and worse CSS (*P*<0.001) than a PLNR < 13%. IPTW-adjusted Kaplan-Meier analysis showed a similar result (OS: *P*<0.001 in the IPTW-adjusted log-rank test; CSS: *P*<0.001 in the IPTW-adjusted log-rank test) (**Figure 4**). In the univariate Cox proportional hazards regression analysis, a PLNR ≥13% was significantly associated with worse OS (HR = 3.364, 95% CI 2.097- 5.395, *P*<0.001) and worse CSS (HR = 7.874, 95% CI 4.247-14.6, *P*<0.001). In the IPTW-adjusted Cox proportional hazards regression analysis, a PLNR ≥13% was an unfavorable risk factor for OS (HR = 2.358, 95% CI 1.1- 5.054, *P* = 0.0275) and CSS (HR = 8.190, 95% CI 2.548- 26.33, *P*<0.001).

### Survival Analyses Before and After IPTW Adjustment: NRN ≥ 13 *vs*. NRN < 13

**Figure 5:**
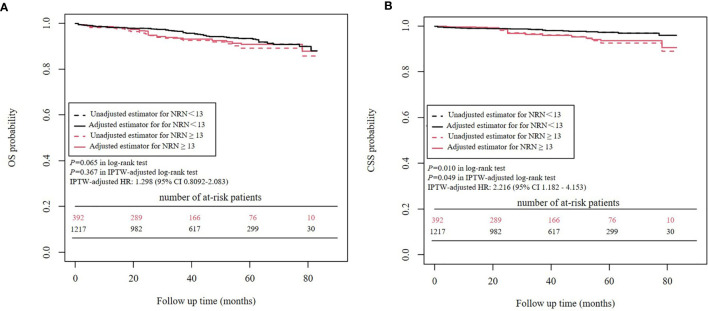
Survival analysis of NRN before and after IPTW. **(A)** OS analysis **(B)** CSS analysis.

Kaplan-Meier analysis and IPTW-adjusted Kaplan-Meier analysis showed that OS was similar between patients with an NRN ≥ 13 versus those with an NRN < 13 (*P*=0.065 in log-rank test and *P*=0.367 in IPTW-adjusted log-rank test) (**Figure 5**). In the univariate Cox proportional hazards regression analysis, NRN ≥ 13 was not significantly associated with worse OS (HR = 1.514, 95% CI 0.9709- 2.362, *P*=0.0673). In the IPTW-adjusted Cox proportional hazards regression analysis, NRN ≥13 was still not a significantly unfavorable risk factor for OS (HR = 1.298, 95% CI 0.8092-2.083, *P* = 0.279).

Kaplan-Meier analysis and IPTW-adjusted Kaplan-Meier analysis showed that an NRN ≥ 13 was significantly associated with worse CSS (*P*=0.010 in log-rank test and *P*=0.049 in IPTW-adjusted log-rank test) (**Figure 5**). In the univariate Cox proportional hazards regression analysis, an NRN ≥ 13 was significantly associated with worse CSS (HR = 2.216, 95% CI 1.182 - 4.153, *P=*0.013). The IPTW-adjusted Cox proportional hazards regression analysis revealed a similar result (HR = 2.162, 95% CI 1.106- 4.226, *P=*0.024).

## Discussion

Lymph node dissection or sampling is of important significance due to the potential staging and therapeutic benefits. Guidelines, including that of the NCCN, ENETS, NANETS, and ESMO, have recommended LND and LND has become commonly applied during PC surgery (4–6). However, there are some unmet needs to be addressed. The role of lymph node involvement status in prognosis needs to be discriminated in more detail than positive/negative status. In addition, the therapeutic benefit of more extensive LND, which could be simply assessed by the NRN, remains unclear. In this IPTW-adjusted analysis, a higher PLNR was a predictor for poorer survival; more extensive LND was not associated with improved OS of patients with PCs but had an adverse influence on CSS.

Lymph node involvement is prevalent in patients with PCs, in particular those with ACs. Pathologically positive lymph nodes could be identified in up to 27% of patients with TCs and in up to 47% of those with ACs, and lymph node involvement has been suggested to be an independent predictor for the prognosis of patients with PCs (7, 17–22). The ratio of positive to examined lymph nodes, PLNR, has been proposed to provide more detailed information regarding the extent of lymph node involvement. PLNR has been shown to be an important prognostic factor in several malignancies, including breast, colorectal and gastric cancer (23–25). It also performed well in the stratification of other NETs, including gastric, small intestinal and pancreatic NETs (9–11). In this study, in survival analyses both before and after the IPTW adjustment, a PLNR ≥13% was proposed for the first time as the cut-off value to predict prognosis and was significantly associated with poor OS and CSS. The currently applied TNM classification for lung cancer was originally designed for non-small cell lung cancer (NSCLC), and it has been validated in PCs (1). However, there is still considerable heterogeneity in the outcomes of patients even within the same subcategories, and the TNM classification system mainly focuses on the location of lymph nodes. The PLNR could be an important complement of this classification system.

The NRN is a simple parameter to determine the extent of LND. Although lymph node involvement was detected in a moderate number of patients and a higher PLNR was suggested to be an unfavorable predictor of OS and CSS, more extensive LND did not lead to an improvement in survival. No significant differences were observed in OS either before or after the IPTW adjustment. More unexpectedly, whether IPTW-adjusted or not, an NRN ≥ 13 indicated poorer CSS. In NSCLC, the prognostic value of the NRN has been adequately validated. One study analyzed the optimal NRN using patient data from a Chinese multi-institutional registry (n=5,706) and proposed a threshold of 16 in patients with declared node-negative NSCLC and validated it using the SEER database (n = 38,806). A greater NRN was associated with better long-term survival. Focusing on early NSCLC overall, regardless of the pathological node status, a greater NRN can achieve better OS (8, 26). However, there were neither benefits nor adverse effects on the OS when more extensive LND was performed in patients with PCs. Theoretically, LND might improve survival, as lymphatic metastasis could be partly prevented, but the advantages might be offset by the increased risk for postoperative complications (27). A previous study conducted by Brown et al. showed that LN assessment is not an independent predictor of OS for patients with clinical T1aN0M0 TCs, and an NRN ≥ 10 also brings no benefits (28). This is consistent with our results, in which a cut-off value of 13 was determined by X-tiles with the highest Chi-square score, although both TCs and ACs at all stages were included in our study. In other words, even with the largest differences between the 2 groups, an NRN ≥ 13 did not lead to an improvement in the OS for patients with PCs. However, Chen’s study focusing on ACs showed that LND was a prognostic factor for better OS (13). Since most patients in our study were with TCs, it is reasonable that no benefits were observed in OS among patients with extensive LND (NRN ≥ 13).

No previous studies have been conducted on the relationship between the NRN and CSS in PCs; unexpectedly, we found that regardless of IPTW adjustment, NRN ≥13 appears to be an unfavorable predictor of poor prognosis. Similar results have been presented in gastrointestinal stromal tumors (GISTs) (29). LND led to poorer OS and CSS in GIST patients compared with patients without LND according to their study. Lymph node involvement was not common in GIST patients, and LND might destroy the immune microenvironment of the normal lymph nodes, causing a high risk of recurrence. Their hypothesis could partially explain the unfavorable result in our study. According to previous studies, pathologically positive lymph nodes could be identified in up to 27% of patients with TCs, but most studies reported that lymph node involvement was present in approximately 15% of patients with TCs; thus, lymph node involvement is not as common in patients with TCs as it is in those with ACs. Lymph node involvement was found in approximately 45% of patients with ACs, but ACs only account for 10%-15% of PCs. Overall, approximately 80% of PCs were pathologically N-negative (7, 19, 20). In addition, 85% of patients in our study were at N0 stage. These data indicate that most patients with PCs, especially those with TCs, might not need systematic LND, at least not extensive LND with NRN ≥13, since their disease is at an early stage with no lymph node involvement. In addition, PCs are relatively indolent, slow-progressing tumors (30). Disturbance of the immune microenvironment, which might accompany extensive LND, might adversely facilitate cancer progression, especially for TCs without lymph involvement, which account for most PCs. In addition, unnecessary extensive LND would make more injuries to patients with no survival benefits. 87.5% of patients were with TCs in our study. Therefore, extensive LND with NRN ≥13 might lead to poorer CSS. Futural study could do more subgroup analyses in which TCs and ACs are discussed respectively. Besides the theoretical explanation, some biases might be also introduced into the analysis due to the limitations of SEER database. 3206 patients were excluded from analysis due to missing data, which were appropriately as twice as the included sample (1622 patients). It is difficult to assess the potential biases resulted from such a large number of excluded patients. The result of poorer CSS might be based on this specific sample and external validations are required.

It is worth noting that previous recommendations for LND were based on the incidence of lymph involvement and its prognostic value for poor OS. However, direct evidence of the benefits of LND is limited, as mentioned below. Although the therapeutic effects of LND might be controversial, it plays an important role in staging and predicting prognosis through the lymph node involvement status and PLNR according to this study. There is no evidence that more extensive LND should be performed routinely in PC surgery. Individualized judgement is still recommended. According to the multivariable logistic regression in our study, histology of AC, M1 stage and tumor size ≥2.5 cm could predict a PLNR ≥13%, which can predict poorer outcome, and more frequent follow-up should be applied to detect recurrence early in these patients and adjust the treatment accordingly. To conclude, although more extensive LND might be of limited therapeutic value, assessment of the PLNR could be applied to predict the outcome of PCs, and LND should be considered on an individual basis because of its value for staging.

There are limitations that should be considered in this study. First, only a limited number of patients (1622/8520) were included, as patients were excluded because of multiple malignant tumors, missing data and other reasons. This may have introduced bias into the analysis. Second, although patient data were IPTW-adjusted based on factors that could have influenced outcomes in an attempt to minimize bias, unknown confounders still represent a source of bias. Third, analyses based on the SEER database do not reflect the condition of patients with PC outside of the US. A high-quality prospective randomized trial or a multi-center retrospective study may be of great value to provide further guidance on the extent of LND necessary for patients with resectable PCs.

## Conclusion

Based on the data retrieved from the SEER database, a PLNR ≥13% could predict worse OS and CSS in patients with PCs. Extensive LND with an NRN ≥13 might have no therapeutic value and instead have adverse influence on PC progression.

## Data Availability Statement

The raw data supporting the conclusions of this article will be made available by the authors, without undue reservation.

## Ethics Statement

The studies involving human participants were reviewed and approved by Institutional Review Board of Cancer Hospital Chinese Academy of Medical Sciences. Written informed consent for participation was not required for this study in accordance with the national legislation and the institutional requirements.

## Author Contributions

Conception and design: HZ and JZ. Administrative support: HZ and JZ. Provision of study materials of patients: QC and PW. Collection and assembly of data: All authors. Data analysis and interpretation: QC and ML. Manuscript writing: All authors. All authors contributed to the article and approved the submitted version.

## Funding

This study was supported by the State Key Project on Infection Diseases of China (Grant No. 2017ZX10201021-007-003), the National The capital health research and development of special (2018-1-4021), the National Natural Science Foundation of China (81972311, 82002611), CAMS Innovation Fund for Medical Sciences (CIFMS) (Grant no. 2017-12M-4-002), the Non-profit Central Research Institution Fund of Chinese Academy of Medical Sciences (2019PT310026) and Sanming Project of Medicine in Shenzhen (No. SZSM202011010).

## Conflict of Interest

The authors declare that the research was conducted in the absence of any commercial or financial relationships that could be construed as a potential conflict of interest.
